# From Victim to Minister's Advisor: A Personnal View of Child Protection in France

**DOI:** 10.3389/fped.2021.587806

**Published:** 2021-03-12

**Authors:** Céline Greco

**Affiliations:** Board Member of the French National Council of Child Protection (Conseil National de Protection de l'Enfance, CNPE), Chair of the “Child Health in Child Protection “Task Force”, Department of Neonatal, Pediatric and Adult Pain and Palliative Medicine, Assistance Publique Hôpitaux de Paris (APHP), Necker-Enfants Malades Hospital, Paris, France

**Keywords:** child abuse, denial, lack of knowledge, delay of identification, long-term consequences, healthcare pack

## Abstract

In France, maltreatment of children, that mean “physical, sexual or emotional maltreatment, or neglect of a child” is a very underestimated phenomenon, yet it is running rampant. The various causes of child abuse are subject to denial, and the ensuing delay of identification of victims is profoundly damaging. As a medical doctor and survivor of child abuse, I have a clear vision of the shortcomings of our child protection system. Non-recognition or late recognition of cases of child abuse is directly related to a lack of knowledge and training on this subject, which leads to weaknesses both in terms of detection and reporting of potential cases. Once the children are identified, they need to receive proper care that should not be limited to the social aspects. Indeed, the victims of abuse suffer from important repercussions on their physical and mental health. Without proper care, their health as adults will be severely impacted. These children require specific care and an adapted health care pack, as well as inclusion in the child protection framework. The task force “Child health in child protection” that I manage has suggested the creation of a standardized healthcare protocol for child victims of abuse, with psychological and medical costs fully covered by the Government, especially regarding clinical psychologists and psycho-motor therapists.

## Introduction

Maltreatment of children, defined as any act or series of acts of commission or omission by a parent or other caregiver that results in harm, potential for harm, or threat of harm to a child, is probably the most underestimated and overlooked form of abuse. Worryingly, according to international literature and a recent set of articles published in The Lancet, 10% of children are victims of abuse and neglect in countries with high income (1), representing a major public-health and social-welfare problem. Four forms of maltreatment are widely recognized: physical abuse; sexual abuse; psychological abuse, sometimes referred to as emotional abuse; and neglect.

In France, its assessment is particularly weak and child abuse or neglect is still a taboo subject. There are no reliable statistics concerning the children who are victims of family maltreatment, or even the number of child deaths due to these maltreatments.

As a medical doctor and survivor of child abuse, committing to improve child protection laws means that I can do so with unique insight into the current state of affairs. A close scrutiny of the faults in the system, especially the difficulty of an early identification of the victims as well as the gaps in the management of physical and psychological care, could help to improve the lives of other victims of abuse or neglect.

## Child Abuse or Neglect: How to Improve Lack of Identification and Denial?

In my experience, the weaknesses in the French system are still very obvious in terms of identifying potential victims and taking their health impairment into account. This is due to the lack of awareness of risk factors as well as lack of training in the various professions that come into play.

This unique insight has allowed me to work with the Ministry of Families, Children and Women's Rights in order to create the new law for the protection of children as of the 14th of March, 2016, and to contribute to writing the first action plan against child maltreatment. It has also allowed me to be part of the National Council of Child Protection (*Conseil National de Protection de l'Enfance, CNPE*), an agency run by the prime minister which aims to assist public policy making in terms of child protection, and to manage the ≪ Child health in child protection ≫ task force.

We lack reliable data on the number of children that are victims of family maltreatment in France. This significantly restrains our appreciation of the situation. The absence of statistics also prevents the implementation of preventive policies unlike other fields as road accidents where it is simple to introduce means of prevention (radars, routine control, etc…) and then, assess their impact by observing the variation of the rate of deaths.

The “CNPE” established in January 2017, has recommended first and foremost to set in motion a commission for the development of research in child protection.

Whether it is physical, sexual or psychological, abuse exists in all social strata and is often very difficult to identify in the most privileged social classes (2).

The difficulty encountered in identifying child abuse or neglect in well-off families is due to the fact that its members will have sufficient knowledge of the system to avoid being identified. Also, it is well-documented that it is difficult for healthcare professionals to report a victim of child abuse that belongs to their same social class (3). Thus, it is particularly difficult to listen to a child whose parent is a lawyer, banker, doctor, or notary. Furthermore, it is often the case that children whose parents belong to higher social classes tend to remain silent, because they assume that they will not be believed if they speak.

As it was in my case. My father was the director of a factory, a respected man. I thought no one would believe my story, and I was afraid to expose my father to rumors and damage his reputation. I worried about what would happen to my mother, who had never worked since my birth, if I exposed my father. How would she manage alone? And all this because of me?

In order to improve identification of victims of child abuse, solid knowledge of risk factors is necessary, such as prematurity or attachment disorder at birth (4). The socio-economical status is irrelevant in such situations. Likewise, specific warning signs (a sharp decline in the child's growth line, bruises on the concave areas of the body etc.) as well as non-specific signs (school absenteeism, behavior disorders, sleep disorders etc.) must be known and lead to further investigations.

The first recommendation of the “CNPE” has been to improve the quality of the training provided to professionals that interact with children; and to ensure that this training is continuous. These training programs are beginning to spread across the national territory, reaching more and more professionals. The aim is to be relevant for a pluri-professional public so that the various actors involved, including doctors, lawyers, social workers, can learn to work together. It has to be noticed that France is part of the ERICA training programme (https://projects.tuni.fi/erica), a project funded by the European Union's Rights, Equality and Citizenship Programme, that aim to develop a training program for professionals to enhance their use of risk assessment tools and their engagement in multiprofessional working practices, to integrate a strategy which involves the civil society in the prevention of child maltreatment, to develop a training program and a mobile application on risk assessment also for parents in order to improve prevention of child maltreatment in Europe.

In addition to the fact that professionals must be better trained in identifying victims of child abuse or neglect, we must also change the paradigm regarding our expectation of an alert that could come from the child himself

Too often, the weight of speaking out rests on the child's shoulders only. They are invited to confide, to dial helplines, to speak…

This shift of responsibility from the adults to the child fails to take into account important factors such as the psychological pressure a parent may put on a child, the child's emotional dependence on the parent, the guilt that may arise from risking harm on the family, as well as the solitude and suffering the child will progressively grow accustomed to. All these factors will prevent the child from spontaneously speaking out.

Furthermore, one must take into account that victims of child abuse or neglect will often have brothers and sisters, who may or may not be spared from maltreatment. Thus, it is important to interview the siblings too.

In my case, despite a substantiated report of child abuse, my younger sister was never taken into account in the procedure, and she was never interviewed. Her status as a victim, a witness to violence and its after-effects, was never taken into account.

As a doctor in the emergency room, I have taken care of children who were victims of maltreatment by one or both their parents. While they were in my care, I sometimes found out that their siblings had already been placed in foster families, and that reports had been made concerning these specific children, but that the brothers and sisters who had not been mentioned in those reports were still living with their parents and their situation had never been taken into account.

Due to this, I fought for thefollowing article to be added to the law of March 14th, 2016 that stipulates that “*the assessment of the situation of a minor following a report of maltreatment is carried out by a multidisciplinary team of specially trained professionals. While this is carried out, the situation of the other minors present in the home is also assessed*.” (article 9).

## The Absolute Need to Take Into Account the Physical and Psychological Health of Child Victims of Abuse

If we can retain the progress allowed, in France, by the law of March 14, 2016, it is necessary to highlight the article 21 allowing the creation of a “life plan for the child” that aims to guarantee its physical, psychological, emotional, intellectual and social development.

This “life plan” includes for the first time a medical and psychological assessment of the child in order to detect the care that he needs to receive to ensure his proper developpment.

Indeed, the children victims of abuse and/or neglect suffer important repercussions on their physical and mental health. Maltreatment experienced during childhood leads to abnormally high death rates; trauma; delays in growth of sensory and cognitive developments; and impaired psychological, emotional, and social development (5–8). There is also a higher risk of aggression (against themselves or others) and delinquency.

When these children become adults, it is not uncommon for them to have developed either a mental, physical or social impairment. There is also an abnormally high rate of them suffering from a chronic disease (e.g., obesity, cardiovascular events, strokes, cancer) (9, 10).

In the USA, the estimated health cost for these patients is three times higher now that they are adults than if these issues had been addressed during their childhood (11).

The children and teenagers currently taken in charge by the child welfare system are at a higher risk of developing psychiatric illness and speech defect. They also suffer from a lack of opportunity, having less access to specialized care such as speech therapy.

Despite all the evidence, only 35% of children included in the French Social Aid for Children receive a systematic health check, and just 53% of the youth in the care of the Judiciary Protection for Juveniles (12).

The demographic crisis concerning pedopsychiatry in France, the absence of financial aid to healthcare professionals, as well as the current policy of somatic and psychological care of minor victims do not allow for an efficient health path.

In my personal situation, I was never given the opportunity to see a doctor during all the time I spent in foster homes. I was never given health checks although I weighed 33 kg (72Lb) at 14 years old and had obvious signs of dietary deficiencies.

A French report, issued on the 28th of February 2017, stated that a consensus had been reached on the need to develop and reinforce provision of the fundamental needs of children. The Child Care Agency defines these universal needs, pointing out a *meta-need* for security, specific needs in situations of impairment, and needs concerning the consequences of adverse childhood experiences.

The task force that I manage ≪ Child health in child protection ≫ has suggested, in accordance with the Decree no 2017-1866 of December 29th 2017 regarding the defining of a national strategy for the 2018-2022 time period, the instauration of a specialized pediatric and pedopsychiatric health path for child and teenage victims of abuse or neglect, whether they live with their families or with the Child Care Agency.

This health path program has been accepted by the State Secretary responsible for child protection and has been strongly supported by France's First Lady: Mrs. Brigitte Macron. It will be carried out by trained healthcare professionals (in both the public and private sector). The program will be supported by referent hospitals with pluridisciplinary pediatric and pedopsychiatric centers that are specialized in child protection. This will be done in collaboration with local referring doctors in child protection, maternal, and child health services, school health services, medicospychological centers, as well as other medico-social organizations.

This health path should be put into place soon. Psychological and medical costs should be fully covered by the Government, especially regarding clinical psychologists and psycho-motor therapists. These costs will most likely be supported by the regional public health authorities.

## Conclusion

Several mountains still have to be moved before we can improve the fate of children who are victims of maltreatment within their family. I believe that our society and the governing authorities are now ready to take action. Indeed, the “PACTES Enfance” plan will be launched shortly, due to the collective effort of several French institutions. This plan will soon be carried out in the Ile de France Region, which is inhabited by over 3 million children. We hope that it will then be extended to the entire French territory. The plan has two main goals: the first is to create multiple referral teams of child protection in our pediatric hospitals, so as to improve identification and care of child victims; the second goal is to create the “PACTES Enfance Federation,” whose aim is to facilitate access to care for children and adolescents who are supported by welfare services, as well as to coordinate the various health professionals who will work with them. Correctly handling the health of child victims allows for reduction of both financial and human costs. Indeed, this allows for more stable placements and less need for psychiatric care and specialized education. We can also anticipate lesser costs during adulthood: less legal procedures, less physical and mental impairment, less need for social assistance and overall improved employability.

## Data Availability Statement

The original contributions presented in the study are included in the article/supplementary material, further inquiries can be directed to the corresponding author/s.

## Author Contributions

The author confirms being the sole contributor of this work and has approved it for publication.

## Conflict of Interest

The author declares that the research was conducted in the absence of any commercial or financial relationships that could be construed as a potential conflict of interest.

## References

[1] Gilbert R Widom CS Browne K Fergusson D Webb E Janson S. Burden and consequences of child maltreatment in high-income countries. Lancet. (2009) 373:68–81. 10.1016/S0140-6736(08)61706-7 19056114

[2] Tursz A, Les oubliés. Enfants maltraités en France et par la France, Editions du Seuil (2010).

[3] Flaherty EG Jones R Sege R. Telling their stories: primary care practitioners' experience evaluating and reporting injuries caused by child abuse. Child Abuse Neglect. (2004) 28:939–45. 10.1016/j.chiabu.2004.03.013 15450760

[4] Paavilainen E Finck A. Effective Methods for Identifying Child Maltreatment in Social and Health Care. Hotus Clinical guideline (2015). Available online at: https://www.hotus.fi/wp-content/uploads/2019/03/maltreatment-hs-sum-eng.pdf

[5] Silverman AB Reinherz HZ Giaconia RM. The long-term sequelae of child and adolescent abuse: a longitudinal community study. Child Abuse Neglect. (1996) 20:709–23. 10.1016/0145-2134(96)00059-2 8866117

[6] Bronsard G Alessandrini M Fond G Loundou A Auquier P Tordjman S. The prevalence of mental disorders among children and adolescents in the child welfare system: a systematic review and meta-analysis. Medicine. (2016) 95:e2622. 10.1097/MD.0000000000002622 26886603 PMC4998603

[7] Rousseau RE Rozé D Duverger M Saulnier P. Devenir à long terme de très jeunes enfants placés à l'Aide Sociale à l'Enfance. Rev Franç Aff Soc. (2016) 5:343–74. 10.3917/rfas.161.0343

[8] Kaiser C. Facteurs de risque psychosociaux et troubles psychiatriques des jeunes pris en charge par l'Aide Sociale à l'Enfance et ayant recours à des soins hospitaliers Neuropsychiatr l'Enfance l'Adolesc. (2011) 59:393–403. 10.1016/j.neurenf.2011.07.001

[9] Norman RE Byambaa M De R Butchart A Scott J Vos T. The long-term health consequences of child physical abuse, emotional abuse, and neglect: a systematic review and meta-analysis. PLoS Med. (2012) 9:e1001349. 10.1371/journal.pmed.1001349 23209385 PMC3507962

[10] Barreyre JY. OBSERVATOIRE NATIONAL DE L'ENFANCE EN DANGER. Une souffrance maltraitée. Parcours et situations de vie des jeunes dits ≪ incasables ≫. Paris: ONED (2008). 10.3917/inso.156.0080

[11] Fang X Brown DS Florence CS Mercy JA. The economic burden of child maltreatment in the United States and implications for prevention. Child Abuse Neglect. (2012) 36:156–65. 10.1016/j.chiabu.2011.10.006 22300910 PMC3776454

[12] Séverine Euillet PM. Juliette Halifax, Nadège Séverac., L'accès à la santé des enfants pris en charge au titre de la protection de l'enfance: accès aux soins et sens du soin. Paris (2016).

